# Psoriasis and Cardiovascular Risk: Assessment by CUORE Project Risk Score in Italian Patients

**DOI:** 10.1155/2013/389031

**Published:** 2013-09-08

**Authors:** Spyridoula Doukaki, Valentina Caputo, Maria Rita Bongiorno

**Affiliations:** Department of Dermatology, University of Palermo, Via del Vespro 131, 90123 Palermo, Italy

## Abstract

*Background*. Psoriasis is a common inflammatory and immune-mediated skin disease. There is growing controversy as to whether cardiovascular risk is elevated in psoriasis. A number of studies suggest a high prevalence of cardiovascular risk factors as well as cardiovascular diseases in psoriasis patients. *Objective*. The objective of this study was to estimate cardiovascular risk score in psoriasis patients and the relation between cardiovascular risk and psoriasis features. Cardiovascular risk was assessed by CUORE project risk score built within the longitudinal study of the Italian CUORE project and suited to populations with a low rate of coronary heart disease. *Results*. A case-control study in 210 psoriasis outpatients and 111 controls with skin diseases other than psoriasis was performed. CUORE project risk score was higher in patients than controls (6.80 ± 6.34 versus 4.48 ± 4.38, *P* < 0.001). Compared to controls, psoriasis patients have higher risk of developing major cardiovascular events. Cardiovascular risk was not related to psoriasis characteristics. *Conclusion*. Increased focus on identifying cardiovascular risk factors and initiation of preventive lifestyle changes or therapeutic interventions in patients with psoriasis is warranted.

## 1. Introduction

Psoriasis is a common inflammatory, immune-mediated skin disease [1]. It affects approximately 2-3% of the Western population, with varying prevalence among different ethnic groups and a similar prevalence in men and women [2]. Although psoriasis can present at any age, it has two peak periods of onset: at 15 to 25 years of age (type I psoriasis) and at 40 to 60 years of age (type II psoriasis) [3]. 

In addition to its cutaneous manifestations, psoriasis has been associated with inflammatory arthritis, depression, anger, anxiety, frustration, and considerable health-related quality of life impairment, which appear independent of objective disease severity [4, 5].

Once symptoms occur, the disease is characterized by a chronic course; spontaneous, long-term remissions occur in a minority of patients. Clinical manifestations are heterogeneous, ranging from limited to very extensive disease [1]. Approximately 80% to 85% of patients have limited skin involvement, whereas 15% to 20% have more extensive skin involvement that may require systemic therapy [6]. 

The exact etiology of psoriasis is not entirely elucidated; there is strong evidence that the interaction of multiple genetic, immunologic, and environmental factors contribute to its pathogenesis. As the understanding of psoriasis has evolved, so has the perception of disease pathophysiology [1, 7], characterized by increased T lymphocyte activity [6]. T-helper (Th)-1, Th-17, and Th-22 cell populations are expanded and stimulated to release inflammatory cytokines (i.e., tumour necrosis factor-*α* (TNF-*α*), interleukin- (IL-) 17, and IL-22) demonstrating the extent of systemic involvement. Accordingly, the inflammation that drives psoriatic pathology is systemic [1]; this concept carries important public-health implications and has prompted a growing body of research. As a systemic inflammatory condition, psoriasis may be analogous to other inflammatory, immune disorders, such as systemic lupus erythematosus and rheumatoid arthritis. Since the risk of myocardial infarction and other cardiovascular diseases is firmly established in these specific disorders, attention has been focused on whether cardiovascular risk factors and cardiovascular diseases are increased in patients with psoriasis [4, 8]. 

Although, the initial link between psoriasis and coronary artery disease was suggested in the 1970s [9, 10], there is growing controversy as to whether cardiovascular risk is elevated in psoriasis. A number of studies suggest a high prevalence of cardiovascular risk factors (e.g., smoking, diabetes mellitus, hypertension, and hyperlipidemia) as well as cardiovascular disease (CVD) in psoriasis patients [6]. A major limitation of most of these studies is that they focus on highly selected psoriasis patients, such as those hospitalized for their disease and are therefore likely biased toward patient populations with more severe disease. This selection bias may be important since some studies indicate that the increased cardiovascular risk may be confined to patients with severe skin disease [6].

Current recommendations on the prevention of coronary heart disease in clinical practice stress the need to base intervention on an assessment of the individual's total burden of risk rather than on the level of any particular risk factor. This is because most people who develop atherosclerotic cardiovascular disease have several risk factors which interact to produce their total risk. It follows that there is a need for clinicians to be able to estimate total risk of cardiovascular disease [11].

In contrast to the general population and patients with diabetes, rheumatoid arthritis, and systemic lupus erythematosus, there are very few information about the estimation of cardiovascular risk by means of specific risk scores in psoriasis patients using predictive equations [12]. Recent studies have examined the risk of cardiovascular events in patients with psoriasis according to the Framingham cardiovascular risk prediction score and documented that a high proportion of patients with psoriasis were at substantially increased risk and making them potential candidates for pharmacological cardiovascular primary prophylaxis [8]. The Framingham risk score is a tool to predict the absolute risk of major coronary and cerebrovascular events at 5 and 10 years in adults from 30 to 74 years of age by stratifying patients into 3 risk categories: patients scoring less than 10% are at low risk, those between 10% and 20% have a moderate risk, and those scoring 20% or more are at high risk. This score is appropriate for United States, Australia, and New Zealand populations [11]. 

Until the recent publication of the Italian risk charts of the “CUORE project,” the Framingham risk function was applied for the Italian population. However, it is known that the Framingham function-based risk charts generally tend to overestimate absolute risk in populations with a low rate of coronary heart disease, such as Italy and, sometimes, this overestimation may occur in countries with a high rate [13]. Moreover, recent studies applying Framingham risk function to data from Danish and German prospective studies have demonstrated that the Framingham risk function clearly overestimates coronary heart disease risk also in these populations [11]. 

The objective of the current study was to estimate cardiovascular risk score in psoriasis patients and the relation between cardiovascular risk and psoriasis features in a real-world setting. 

## 2. Materials and Methods

An observational case-control study was performed at the psoriasis outpatient clinic of Dermatology Department (University of Palermo, Italy), over a period of 1 year from January 2012 through December 2012. The control group was recruited from nonpsoriatic patients attending the same Dermatology Department and included patients with melanocytic naevi, cutaneous melanoma, nonmelanoma skin cancer, cutaneous infectious diseases, and other benign conditions. Patients suffering from inflammatory skin conditions or autoimmune diseases were excluded from the control group. Informed consent was obtained from all patients prior to inclusion and confidentiality of personal data was warranted. 

The 10-year cardiovascular risk was assessed using the CUORE project risk score built within the Italian CUORE project. The CUORE project—epidemiology and prevention of ischaemic heart diseases—launched in 1998, is financed by 1% of the National health fund and is coordinated by the Istituto Superiore di Sanità. It is a prospective fixed-cohort study, including cohorts from the north, the centre, and the south of Italy. Since 2005, the project is listed among those of the National Centre for Disease Prevention and Control, Ministry of Health, Rome.

The CUORE project risk score allows estimating the probability of experiencing a first cardiovascular event (myocardial infarction, stroke) over the next 10 years knowing the level of eight risk factors for cardiovascular disease: age, gender, systolic blood pressure (SBP), total blood cholesterol (TC), high-density lipoprotein-cholesterol (HDL-C), diabetes mellitus, smoking habit, and use of antihypertensive medication. It is validated in patients 35 to 69 years of age and without previous major cardiovascular accidents. Mathematical functions, derived from longitudinal studies carried out on population groups followed up over time, are used to assess the risk score. On the basis of the CUORE project risk score, patients are classified as low risk (CUORE project risk score < 3.0%), risk to be kept under control through the adoption of a healthy lifestyle (CUORE project risk score 3.0–19.9%), and high risk (CUORE project risk score ≥ 20.0%). 

Data were collected using the software cuore.exe, downloadable free of charge from the website of the CUORE project http://www.cuore.iss.it/. Information recorded were cigarette smoking, personal history of myocardial infarction, stroke, hospitalization for major cardiovascular events and medication use, and clinical type and duration of psoriasis. 

Risk factors were assessed by using standardized procedures. Laboratory analyses considered for risk assessment were TC, HDL-C, and glycaemia. Participants were defined as having diabetes mellitus when they were taking hypoglycaemic medications (oral hypoglycaemic drugs or insulin), had a fasting plasma glucose concentration ≥ 126 mg/dL, or had a personal clinical history of diabetes.

Patients and controls blood pressure was assessed by trained technicians before their routine dermatologic followup at the Dermatology Department of University of Palermo. Blood pressure was measured twice on the right arm, using a mercury sphygmomanometer, with the participant sitting after resting for 5 minutes. Systolic and diastolic blood pressure was recorded (first and fifth Korotkoff sounds); the average of two measurements, taken 5 min apart, was used as the study variable.

Inclusion criteria for psoriasis patients were a diagnosis of psoriasis lasting at least 1 year; absence of any clinical sign and symptom of articular involvement or diagnosis of arthropathy; absence of any systemic treatment for psoriasis in the previous 3 months prior to clinical evaluation. The severity of the psoriasis was measured by means of the Psoriasis Area and Severity Index (PASI), the most commonly used tool to assess disease severity in patients with psoriasis in clinical trials. It measures erythema, infiltration, scaling, and extent of involvement of the four body areas (head, trunk, arms, and legs). The PASI scale ranges from 0 to 72. Psoriasis is classified as mild if the PASI is below 10 and moderate to severe if it is 10 or above [14, 15]. Patients were considered to have early-onset psoriasis if the onset age was <40 years.

Data management and statistical analysis were carried out using WINKS SDA, version 7.0. Qualitative variables were described as absolute and relative frequencies while quantitative variables were described using means ± standard deviation (SD). *P* values < 0.05 were accepted as statistically significant. The analysis was performed for the sample as a whole and for subgroups of patients classified according to disease severity (mild or moderate to severe) and type (type I or type II psoriasis).

## 3. Results

The study included 210 patients and 111 controls. Mean age of patients with psoriasis was 52.22 ± 9.85 years (mean ± SD) and 142 (67.62%) were male. Disease severity was assessed according to the psoriasis area and severity index. Seventy (33.3%) patients had mild disease and 59.05% (124) had type I psoriasis. The mean PASI was 15.44 ± 8.67 (range 4–46.7). Psoriasis duration was 1 to 54 years (mean 14.18 ± 10.93).

The mean age of the nonpsoriatic group was 52.30 ± 9.55 years and 75 (67.57%) were male. Mean age (*P* = 0.994) was similar in cases and controls. There were no significant differences in gender between the groups (*P* = 0.992). Seventy (63.06%) controls had nonmelanoma skin cancer, melanocytic nevi, or cutaneous melanoma and 36.94% (41 controls) had cutaneous infectious diseases, such as dermatophytosis, onychomycosis, candidiasis, pityriasis versicolor, warts, condylomata, exanthematous viral diseases, or leishmaniasis. Descriptive analyses of the demographic characteristics of patients and controls appear in Table 1.

CUORE project risk score was calculated in patients and controls and it was higher in patients than controls (mean ± SD, 6.80 ± 6.34 versus 4.48 ± 4.38, *P* < 0.001). On the basis of the CUORE project risk score, 87 (41.43%) psoriasis patients had a low risk of suffering a major cardiovascular event in ten next years, 112 (53.33%) an intermediate risk, and 11 (5.24%) a high risk, compared with 46.85% (52) (low risk) and 53.15% (59) of subjects (intermediate risk) in the control group (Table 2). In the low risk group, CUORE project risk score mean was higher in patients than controls (1.81 ± 0.74 versus 1.28 ± 0.6, *P* < 0.001). Mean age of patients (45.02 ± 6.79) and controls (45.56 ± 7.43) with a low risk score was similar (*P* = 0.662). Evenly, in the intermediate risk group, patients had a higher CUORE project risk score mean than controls (8.9 ± 4.25 versus 7.30 ± 4.43, *P* = 0.002). Mean age of patients (56.6 ± 8.44) and controls (58.25 ± 6.98) with an intermediate risk score was not significantly different (*P* = 0.063). We did not observe controls with a high risk score. 

Patients with type II psoriasis had a higher CUORE project risk score, but it could be because of a higher mean age (*P* < 0.001). Indeed, CUORE project risk score mean was no not significantly different in all age groups in type I and II psoriasis (Table 3). There was no correlation between psoriasis severity and CUORE project risk score (Table 4).

## 4. Discussion

Psoriasis has been associated with an increased risk of metabolic syndrome and its components (diabetes mellitus, hypertension, hyperlipidemia, obesity, and smoking). Diagnosis of metabolic syndrome carries with it an increased risk of cardiovascular morbidity and mortality and has important public-health implications [16].

The majority of epidemiological studies examining the cardiovascular risk in psoriasis relied on Framingham risk score [19–22] and one assessed DORICA, SCORE, and REDIGOR risk score [12]. Framingham risk score is considered to overestimate cardiovascular risk in European countries with a lower incidence of cardiovascular events while DORICA, SCORE, and REDIGOR are more suited to Mediterranean populations [12]. 

In our series, the 10-year CUORE project cardiovascular risk score was assessed to patients and controls. Consistent with previous research, this study found a greater cardiovascular risk score in psoriasis patients than controls. However, we report a higher proportion of patients with moderate cardiovascular risk (53.33%) while a minority of patients (5.24%) were at high risk of cardiovascular disease. We posit that this was due to the overestimation of Framingham cardiovascular risk score in European populations. According to previous research, we found that cardiovascular risk increases with age. 

There is growing controversy as to whether cardiovascular risk is elevated in patients with moderate to severe psoriasis or those who had an earlier age of disease onset. Some published studies indicate that the increased cardiovascular risk may be confined to patients with severe skin disease [17]. However, studies that focused on patients hospitalized for psoriasis may not be generalizable to the broader population of patients with psoriasis because only a few patients with the most severe disease require hospitalization. Furthermore, patients hospitalized for any condition generally have higher rates of comorbidities, smoking, and alcohol use, which can increase the risk of death compared with individuals who are not hospitalized [18]. 

In our series, consisted of 210 psoriasis outpatients, which reflect the complete disease spectrum, course, and severity, no relationship was found between disease severity and cardiovascular risk score. Moreover, there was no correlation between psoriasis type and cardiovascular risk; this relation was not evaluated in previous studies. In a previous study, no correlation between disease duration and Framingham risk score has been reported [19]. Our findings, although the number of psoriasis patients and controls are limited, demonstrate that cardiovascular risk is higher in psoriasis even when assessed by a risk score not previously used in psoriasis patients and more suited to Mediterranean populations. 

The aim of cardiovascular disease risk charts is to be a simple diagnostic and easily usable support in the clinical practice of general practitioners and specialists. They describe the disease risk in a population much better than using any single risk factor. Ten-year cardiovascular risk assessment can be the first step to implement preventive actions in primary care.

Several studies [12, 19–22] and our findings demonstrate that cardiovascular diseases and their associated risk factors are more common in patients with psoriasis than in the general population. The cause of this elevated risk is unclear. Some authors suggest that the profound psychological impact of psoriasis may drive risky behaviours such as obesity and smoking and thereby directly increase cardiovascular risk [23]. Others have suggested that there may be some intrinsic associated risk: elevated lipids have been documented in psoriasis patients at the time of their initial diagnosis when compared to nonpsoriasis controls that were matched for body mass index (BMI) status, as well as other demographic, clinical, and lifestyle characteristics. However, the explanation of the association between psoriasis and cardiovascular disease is likely to be more complex and multifactorial [10]. New evidence has led to the hypothesis that psoriasis confers increased cardiovascular risk above and beyond traditional risk factors. Psoriasis and atherosclerosis are chronic inflammatory diseases with a considerable overlap of inflammatory mechanisms [24]. Involvement of T lymphocytes in psoriasis immunopathogenesis, linked to Th1 and Th17 patterns of immunological response, is believed to lead to a proinflammatory state, which has been associated with an increased risk of cardiovascular disease [20, 25]. 

Recognizing the increased prevalence of cardiovascular disease, the National Psoriasis Foundation has issued a consensus statement that alerts providers that patients with psoriasis may represent an emerging high-cardiovascular risk population and thus patients with psoriasis should be screened for cardiovascular risk factors. This consensus statement further recommends that appropriate lifestyle and pharmacologic therapies should be prescribed for patients with psoriasis who are at increased risk for cardiovascular diseases [5]. Of note, it has recently been suggested that patients with psoriasis are inadequately screened and treated for coronary risk factors. Underdiagnosis and undertreatment of coronary risk factors may contribute to the increased risk of cardiovascular disease in psoriasis patients [24]. 

## 5. Conclusion

It is wellestablished that psoriasis is associated with increased risk of cardiovascular disease and increased prevalence of cardiovascular risk factors. Screening practices and treatment aimed at cardiovascular risk factors and disease remain a challenge in clinical practice. It is necessary to enhance dermatologists', cardiologists', internists' and general practitioners' awareness on subclinical atherosclerosis in psoriasis patients, so that general preventive measures and early therapeutic interventions can be implemented reducing the burden of high mortality events such as acute myocardium infarction and stroke. 

Treatment guidelines on management of cardiovascular risk factors and further studies are needed to evaluate the clinical utility of psoriasis in cardiovascular risk prediction and investigate the impact of psoriasis treatment on cardiovascular outcomes.

## Figures and Tables

**Table 1:** Descriptive analysis of the demographic and clinical data of cases and controls.

Characteristics	Psoriasis patients (*n* = 210)	Controls (*n* = 111)	*P* value
Age (years), mean ± SD	52.22 ± 9.85	52.30 ± 9.55	*P* = 0.994
Gender			
Male, % (*n*)	67.62% (142)	67.57% (75)	*P* = 0.992
PASI, mean	15.44 ± 8.67		
Range	4–46.7		
PASI < 10, % (*n*)	33% (70)		
PASI ≥ 10, % (*n*)	77% (140)		
Psoriasis type I, % (*n*)	59.05% (124)		
Psoriasis type II, % (*n*)	40.95% (86)		
CUORE project risk score,			
Mean ± SD	6.80 ± 6.34	4.48 ± 4.38	*P* < 0.001
35–44 years	2.32 ± 1.65	1.11 ± 0.74	*P* < 0.001
45–54 years	4.43 ± 3.47	2.53 ± 1.39	*P* < 0.001
55–69 years	12.99 ± 15.16	8.07 ± 4.68	*P* = 0.006

**Table 2:** Comparison of CUORE project risk score in patients and controls.

	Cases (*n* = 210)	Controls (*n* = 111)	*P* value
Low risk			
% (*n*)	41.43% (87)	46.85% (52)	*P* = 0.351
Age (years), mean ± SD	45.02 ± 6.79	45.56 ± 7.34	*P* = 0.662
Gender			
Male, % (*n*)	22.86% (48)	25.2% (28)	*P* = 0.635
Female, % (*n*)	18.6% (39)	21.6% (24)	*P* = 0.405
CUORE project risk score,			
Mean ± SD	1.81 ± 0.74	1.28 ± 0.69	*P* < 0.001
Intermediate risk			
% (*n*)	53.33% (112)	53.15% (59)	*P* = 0.975
Age (years), mean ± SD	56.6 ± 8.44	58.25 ± 6.98	*P* = 0.063
Gender			
Male, % (*n*)	40% (84)	42.4% (47)	*P* = 0.684
Female, % (*n*)	13.3% (28)	10.8% (12)	*P* = 0.515
CUORE project risk score,			
Mean ± SD	8.90 ± 4.25	7.30 ± 4.43	*P* = 0.002
High risk			
% (*n*)	5.24 (11)	0	
Age (years), mean ± SD	64.73 ± 3.86		
Gender			
Male, % (*n*)	4.77% (10)		
Female, % (*n*)	0.47% (1)		
CUORE project risk score,			
Mean ± SD	24.82 ± 3.19		

**Table 3:** Comparison of psoriasis type and cardiovascular risk.

	Psoriasis type I (*n* = 124)	Psoriasis type II (*n* = 86)	*P* value
Age (years), mean ± SD	48.5 ± 9.22	57.6 ± 8.23	*P* < 0.001
Gender			
Male, % (*n*)	72.6% (90)	60.5% (52)	*P* = 0.065
Female, % (*n*)	27.4% (34)	39.5% (34)	
CUORE project risk score,			
Mean ± SD	5.36 ± 5.41	8.87 ± 6.99	*P* < 0.001
35–44 years	2.44 ± 1.71	1.5 ± 0.76	*P* = 0.023
45–54 years	4.75 ± 3.71	3.83 ± 2.97	*P* = 0.319
55–69 years	11.27 ± 6.89	11.72 ± 6.78	*P* = 0.771

**Table 4:** Comparison of psoriasis severity and cardiovascular risk.

	PASI < 10 (*n* = 70)	PASI ≥ 10 (*n* = 140)	*P* value
Age (years), mean ± SD	52.5 ± 10.3	52.1 ± 9.69	*P* = 0.783
Gender			
Male, % (*n*)	71.43% (50)	65.71% (92)	*P* = 0.404
Female, % (*n*)	28.57% (20)	34.29% (48)	
CUORE project risk score,			
Mean ± SD	6.91 ± 6.75	6.74 ± 6.14	*P* = 0.885
